# Permanent Deformation and Stiffness Degradation of Open Hole Glass/PA6 UD Thermoplastic Composite in Tension and Compression

**DOI:** 10.3390/ma14102646

**Published:** 2021-05-18

**Authors:** Ruben Dirk Sevenois, Xiaoyu Yang, Erik Verboven, Mathias Kersemans, Wim Van Paepegem

**Affiliations:** 1 Department of Materials, Textiles and Chemical Engineering, Faculty of Engineering and Architecture, Ghent University, Technologiepark Zwijnaarde 46, 9052 Zwijnaarde, Belgium; xiaoyu.yang@ugent.be (X.Y.); Erik.Verboven@UGent.be (E.V.); Mathias.Kersemans@UGent.be (M.K.); Wim.VanPaepegem@UGent.be (W.V.P.); 2 The State Key Laboratory of Fluid Power Transmission and Control, Zhejiang University, Hangzhou 310027, China

**Keywords:** glass fibers, PA6, fiber-reinforced thermoplastic composite, digital image correlation, plastic deformation, mechanical testing, tension, compression, ultrasonics

## Abstract

UD glass/PA6 coupons with an open hole are subjected to tensile and compressive loading. Three layups: [0/90]_5s_, [+45/−45]_5s_ and [+45/0/−45/90]_3s_ with a shape based on ASTM D5766 were tested. Both monotonic loading as well as loading–unloading–reloading tests were executed. The strain field on the sample surface was measured with digital image correlation. This allowed identifying the distribution of the strain field during loading, permanent deformation and the evolution of the sample elastic modulus. This information is not frequently measured. Yet, it is vital for the development and validation of advanced failure models. The results indicate that the thermoplastic matrix allows large plastic deformation under tensile loading for the specimens with layup [+45/−45]_5s_. In addition, the specimen elastic modulus reduces by about 70%. The other layups show minor permanent deformation, while the elastic modulus reduces by up to 15%. Furthermore, the quasi-isotropic laminate shows a significant post-failure load-bearing capacity under compression loading. The results are complemented with post-mortem damage and fracture observations using optical microscopy and ultrasound inspection.

## 1. Introduction

Technical continuous fiber-reinforced polymers (FRP) where carbon or glass fibers are combined with a thermoplastic (TP) polymer matrix are strong candidates for use in the mass production of composite structures. The TP matrix can be melted, allowing traditional (metal-like) processing techniques, such as hot molding or forming, which have short cycle times. In addition to this, TP-FRP provides improved recyclability and cleaner processing technologies compared to thermoset (TS) FRP [1]. Before TP-FRP can be used in structural design, the mechanical behavior under several loading conditions must be known. This knowledge can then be used to develop and benchmark improved mechanical models.

For TS-FRP [2,3,4], detailed studies about the failure of several combinations of fibers and matrix in the presence of stress concentrations exist. In contrast, for TP-FRP, only a limited amount of studies are available. The effect of the hole drilling method was investigated by Mariatti et al. [5] for plain woven glass/ABS and Brown et al. [6] for unidirectional (UD) carbon/PEEK. In the studies above, the holes were drilled through the laminate after consolidation, cutting both fibers and matrix. With advanced fiber steering techniques, it is possible to place fibers around hole openings. With this, the fibers are not cut and run continuously through the material. This usually results in an increased notched and bearing strength, as shown in [7,8] for carbon/PA6.

Concerning mechanical behavior and the failure mechanisms occurring in TP-FRP, Liu et al. [9] studied the open-hole compressive (OHC) failure of woven AS4/PEEK. Gavande and Anand [10] studied open-hole tension (OHT) and compression strength of woven E-glass/Elium laminates with a quasi-isotropic (QI) layup. Both report a stiff and brittle response of the TP-FRP laminates tested. Compared to an equivalent TS carbon/epoxy material, Gavande and Anand [10] report a higher shear modulus and shear strength. This is not surprising, considering that 1) these properties are matrix-dominated and 2) the stiffness of the TP matrices are higher. A numerical and experimental study on the notched and unnotched hole biaxial tension and compression strength of UD carbon AS4/PEKK-FC are presented by Vankan et al. [11]. Under both tension and compression, a quasi-brittle response in terms of stress–strain is shown. As the study was directed to obtaining the biaxial failure envelope, an anti-buckling device was used to prevent premature buckling. In addition, digital image correlation (DIC) was used to study the evolution of strain and initiation of damage close to the hole edge. Niitsu and Lopes [12] studied the OHC strength of carbon/PPS after fatigue loading at both room temperature, hot wet, and cold conditions. The study reports decreasing strength and stiffness with increasing temperature and humidity. This is similar behavior when compared to TS carbon/epoxy. Malpot et al. [13] have studied the OHT fatigue strength of twill woven glass/PA66. In their study, an infrared camera was used to study the heating of the material during loading, given identifying damage occurrence. At a frequency of 1 Hz, heating was to about 5 °C at the edge of the hole. The fatigue strength as a function of cycles followed a general Wöhler-like degradation.

The literature shows that most authors focus on retrieving the notched strength under static or fatigue conditions. Except for [11], the strain field (and its evolution) around the hole are not studied. Next to that, glass and carbon fibers are frequently studied as reinforcement fibers. In addition, it is noted that the matrix materials investigated are limited to high-performance polymers like PEEK, PEKK or PPS, which are used in aerospace structures. The properties of TP-FRP with cheaper matrix materials like PA66 or PA6, used in less demanding structures (e.g., automotive applications), are less frequently investigated.

Not only is the demand for the latter materials increasing, but there is also an increasing interest in advanced material models, which can predict their nonlinear behavior. For an in-depth validation of such material models, detailed information is required about the strain field evolution during the loading process. In addition, knowledge of the quantitative evolution of specimen stiffness and permanent strain, which may be caused by a combination of damage phenomena, including matrix cracking and plastic deformation, fiber failure, fiber-matrix debonding and delaminations, is crucial input for such numerical models.

In this work, UD glass/PA6 laminates with an open hole are subjected to tension and compression loading. Both monotonic and loading–unloading–reloading (LUR) tests were executed to identify the evolution of specimen stiffness and developing permanent strain on the specimen surface. This information is rarely reported in the literature. Yet, it is vital to validate advanced material models. In addition, to the authors’ best knowledge, the results of open-hole tests on UD glass/PA6 have not been reported. Additional to the strain field, optical microscopy and transmission ultrasound inspection is used to reveal the main damage areas and damage morphology.

## 2. Materials and Methods

TenCate CETEX UD glass/nylon-6 tape from Ten Cate Advanced Composites B.V. (Nijverdal, The Netherlands) was hot-pressed to laminated plates with layups [0/90]_5s_, [+45/−45]_5s_ and [+45/0/−45/90]_3s_. The composite combines E-glass fibers in a BASF UltraBatch 2400 PA6) resin [14]. The average ply thickness was 0.26 mm. This resulted in a sample thickness of 5.2 mm for the [0/90]_5s_ and [+45/−45]_5s_ layup and 6.2 mm for the QI layup. The [0/90]_5s_ layup illustrates the strength of the [0] plies without splitting since it is prevented by the [90] plies. The [+45/−45]_5s_ layup illustrates the shear performance of the plies. The QI layup is chosen to represent a common layup strategy for laminates from fiber-reinforced polymers.

**Figure 1:**
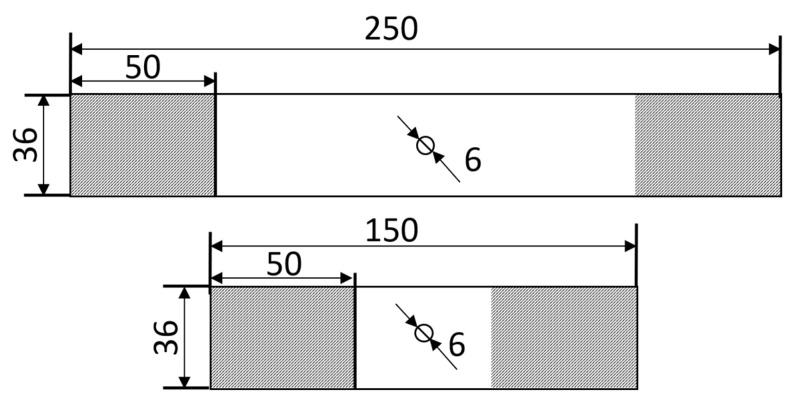
Specimen shapes. **Top**: tensile specimen. **Bottom**: compression specimen. The gray area is the clamped area.

Based on ASTM D5766 [15] and ASTM D6484 [16], rectangular specimens with dimensions 250 mm × 36 mm and 150 mm × 36 mm were cut using water jet cutting. A central hole with a diameter of 6 mm was carefully milled from the coupons. The specimens with 250 mm length were loaded in tension, while the 150 mm long specimens were loaded in compression. The clamping distance was 50 mm. This results in an effective gauge length of 150 mm and 50 mm for the tensile and compressive specimens, respectively. Figure 1 shows the specimen dimensions.

The high thickness is a deviation from the standard. This was deliberately chosen such that the short gauge length combined with the high thickness would prevent, or at least significantly delay, buckling. As such, an anti-buckling guide, which would obstruct the view on the specimen surface to allow identification of the strain field on the sample surface with DIC, is not required. Normally a high thickness is only required under compression. However, since the strength of a laminate can depend on thickness [17], the same laminate thickness was used for the tensile specimens such that both tension and compression loaded cases could be compared.

After production, the specimens were nondestructively inspected to ensure that neither water jet cutting nor hole milling had induced damage to the sample or hole edges. A summary of the specimens and load types is shown in Table 1. The specimens were tested with a 100 kN hydraulic Instron uniaxial test bench () in a climatized room at 23 °C with 50% room humidity (RH). The clamps of the machine were aligned before testing to ensure proper load introduction. Both monotonic loadings until failure and loading–unloading–reloading (LUR) tests were executed. In the LUR tests, consecutive reloadings were done at approximately 25%, 35%, 50%, 65% and 85% of the static strength. After this, the specimen was loaded until fracture.

Force was measured using the load cell of the machine. The strain on the sample surface was measured using 3D DIC. For this, the specimen surface was first painted with white, water-based, acrylic airbrush paint (titanium white, Golden Artist Colors, Inc., New York, USA). This type of paint adheres well to the surface without forming a film. This ensures that the paint does not detach or peel from the specimen surface under large deformation, invalidating the measurement. A black speckle pattern was applied on top of the white surface using matte black-colored spray (Motip matt black acrylic). The 3D DIC system consisted of a custom setup with 2 × 5 megapixels (MP) Pointgrey cameras (Grasshopper 3 GS3-U3-51S5M-C, The images were analyzed using VIC-3D (version 9.1.6) [18], Correlated Solutions.

From the tests, the failure phenomena, the general stress–strain behavior, developing permanent strain and damage (indicated by sample stiffness reduction) are investigated. The failure type of the samples is derived from the images taken with the cameras and side microscopy. For the nominal stress–global strain behavior, the nominal stress, $\sigma_{nom}$, is defined, as shown below:

(1) $$\sigma_{nom\;=\;\frac{F}{\left(w-d\right)\times t}}$$

**Figure 2:**
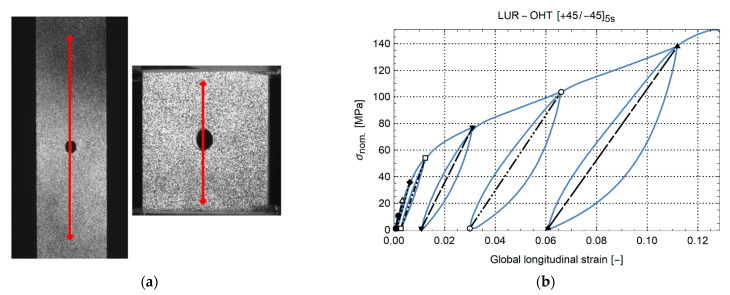
(**a**) Global sample strain gauge for tension (left) and compression (right) specimen, (**b**) nominal stress–global strain of an open-hole sample with layup [+45/−45]_5s_ under tension load.

In Equation (1), F is the force from the load cell, w is the sample width, t is the sample thickness, and d is the hole diameter. The global sample strain is defined as the engineering strain between two points located close to the clamps, as indicated in Figure 2a. This definition indicates the global state of the specimen and allows a representative comparison between the samples. As a result, the forthcoming “stress–strain” curves of these experiments indicate specimen behavior and not material behavior.

The specimen elastic modulus is defined as the slope between consecutive maximum and minimum loads in the unloading–reloading cycle. This is illustrated by the straight black lines in Figure 2b. In the figure, one can also see the development of permanent strain after each loading cycle. After mechanical testing, the specimen sides were polished to identify the fracture mechanisms occurring on the side. Additionally, several specimens were inspected through transmission ultrasound to identify the extent of internal delaminations across the gauge length. The ultrasonic C scans were recorded with an automated scanner system on a rectangular grid. The scan axis measured points with steps of 0.1 mm, while the index axis was moved in steps of 0.5 mm. A through-transmission setup was used with two shockwave transducers with 5 MHz nominal frequency (GE Measurement & Control H5, Rotterdam, The Netherlands) spaced 130 mm apart. For the waveform generation, a UTPR-CC-50 ultrasonic pulser/receiver (Tecscan, Quebec, Canada) with negative spike pulse excitation was used, while data acquisition was handled by a NI-PXIe-5172 card (National Instruments Corporation, Austin, TX, USA). Signals were recorded with a sampling frequency of 125 MS/s. Instrument control was done in LabVIEW (version 2020) [19]. The lateral resolution of the ultrasound is 3.33 mm.

## 3. Results and Discussion

First, the nominal stress–global strain is shown in Section 3.1. Next, in Section 3.2, the observed failure type for each configuration is identified. This is followed by an analysis of the global permanent strain and damage together with.the patterns of permanent strain, as observed by the DIC, in Section 3.3.

### 3.1. Nominal Stress–Global Strain

**Figure 3:**
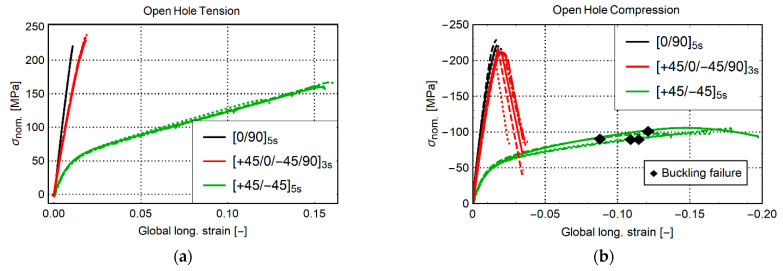
Nominal stress vs. global strain of samples subjected to monotonic loading until failure. (**a**) OHT, (**b**) OHC.

The nominal stress–global strain of the experiments is shown in Figure 3. A different color represents the result for each layup. For the results of samples with the same layup, different dashing is used.

The cases for OHT are shown in Figure 3a. The coupons with layup [0/90]_5s_ and [+45/0/−45/90]_3s_ show quasilinear behavior up to sudden failure at about 220 MPa. The coupon with layup [+45/−45]_5s_ initially shows linear behavior. This, however, turns nonlinear at 1% strain. A failure strain of about 12–15% is observed with a failure load of about 150 MPa. These failure strains are significantly higher than for the other specimens because the +45/−45 plies are primarily loaded in shear. This loading type is matrix-dominated and illustrates the large deformation capability of the PA6 matrix. Remarkable is that the OHT strength of the QI laminate is higher than the OHT strength of the coupon with layup [0/90]_5s_. This is caused by the high OHT strength of the +45/−45 plies, which contribute significantly to the strength of the QI laminate.

**Figure 4:**
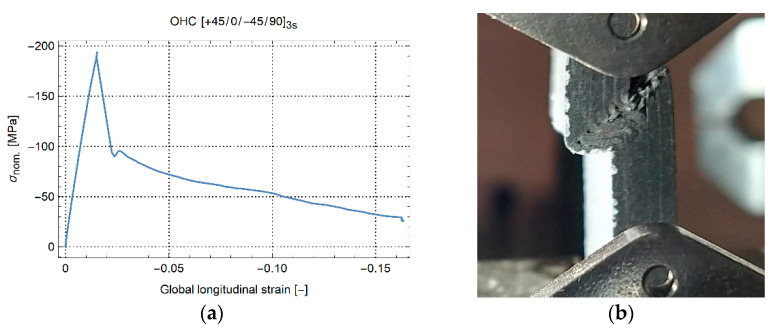
Compression of a specimen with layup [+45/0/−45/90]_3s_. (**a**) Nominal stress–global strain (**b**) side view at −0.16 [-] strain.

The curves for OHC are shown in Figure 3b. Similar to tension, layups [0/90]_5s_ and [+45/0/−45/90]_3s_ show quasilinear behavior up to failure. A small nonlinear region is present close to the final failure. After kinking at maximum load, the QI samples still carried about 35% of the maximum strength at −75 MPa. The curves for layup [+45/−45]_5s_ are nonlinear. A notable difference is that buckling occurs at about −90 MPa, as indicated by the black diamonds. Recall that the end of the curves for [+45/0/−45/90]_3s_ and [+45/-45]_5s_ does not indicate specimen failure. The specimens were removed after, respectively, kinking and buckling to protect the load cell of the machine and preserve the crosshead alignment. Yet, the specimens could still have been compressed more. This was illustrated with the last QI specimen. The result is shown in Figure 4a. After kinking failure, the load-carrying capacity of the material steadily reduces, while the global strain reaches more than 15% global compressive strain. Figure 4b shows the final state of the specimen in the test machine before stopping the test. Both specimen halves were still intact with significant plastic deformation and failure.

### 3.2. Failure Observations

For each of the six combinations of load (tensile and compressive) and layup ([0/90]_5s_, [+45/−45]_5s_, [+45/0/−45/90]_3s_), different failure phenomena were observed.

**Figure 5:**
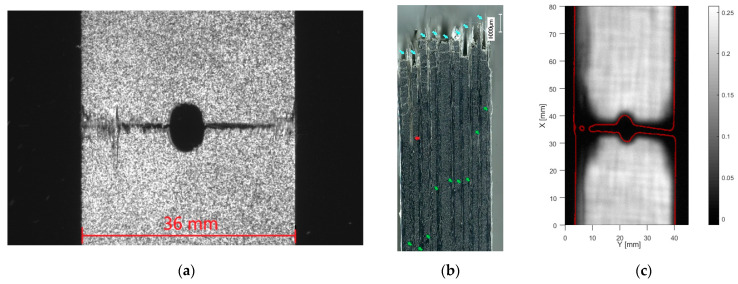
Tensile failure of [0/90]_5s_. (**a**) front view, (**b**) side microscopy, (**c**) through transmission ultrasound (for high resolution, please refer to the online version of this article).

**Figure 6:**
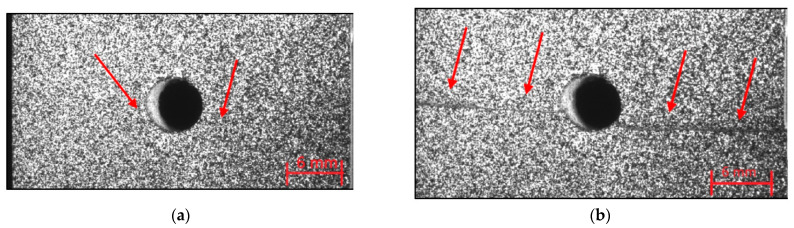

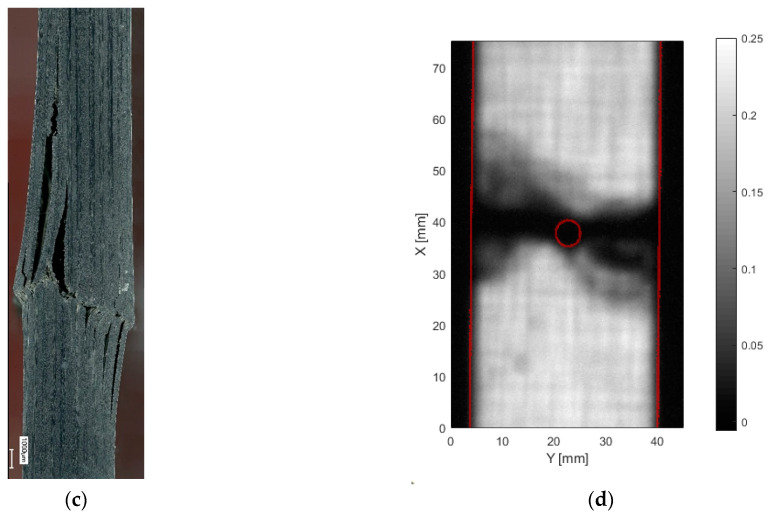
Compressive failure of [0/90]_5s_. (**a**) front view just before failure, (**b**) front view after kinking failure, (**c**) side microscopy, (**d**) through transmission ultrasound (for high resolution, please refer to the online version of this article).

For the samples with layup [0/90]_5s,_ both tensile and compressive loading showed a quasilinear stress–strain behavior with brittle material failure. Figure 5 and Figure 6 show typical failure under, respectively, tensile and compressive load. The videos of the DIC images during loading were analyzed. It is observed that OHT failure of the samples with layup [0/90]_5s_ shows no indication of growing cracks just before sudden failure. In an instant, symmetrical cracks appear from the edge of the holes to quickly span the entire width of the sample. Remarkable for this failure is that several fibers still bridged over the crack at the sides of the specimen. This connection was quite weak yet indicates that next to fiber breakage, fiber pull-out is a damaging phenomenon for this composite material. Microscopic observation of the side of the samples, Figure 5b, shows this. It can be seen that the “pulled-out” fibers (cyan arrows in the figure) continue as ply delaminations as far as 3 mm away from the crack face (red arrow in the figure). Matrix cracking is observed in the [90] degree plies. This is observed at a distance of up to 10 mm from the crack face. The green arrows on the figure indicate the ply cracks observed furthest away from the hole. High-resolution images can be consulted in the online version of the article. Ultrasound inspection, Figure 5c, shows that a limited amount of delamination develops in the hole’s vicinity. The red lines on the figure indicate the edges of the specimen. At the edges of the sample, delaminations are present up to 2 cm away from the crack face.

OHC failure of the samples with layup [0/90]_5s_ shows kinking failure. The samples do not buckle before losing load-bearing capacity. Just before failure, localized kinking can be observed originating from the hole edge. This is indicated by arrows in Figure 6a. Next to that, microscopic observation shows that few matrix cracks developed. Yet, large delaminations occur where plies were pushed apart after kinking failure, Figure 6c. The extent of the internal delamination is illustrated in Figure 6d with ultrasound inspection.

**Figure 7:**
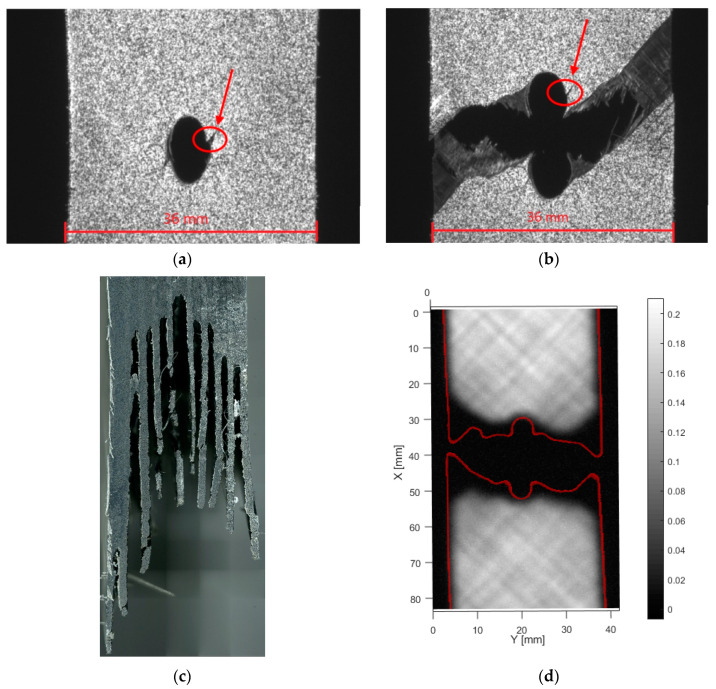
Tensile failure of [+45/−45]_5s_, (**a**) front view just before failure, (**b**) front view after failure, (**c**) side microscopy, (**d**) through transmission ultrasound (for high resolution, please refer to the online version of this article).

**Figure 8:**
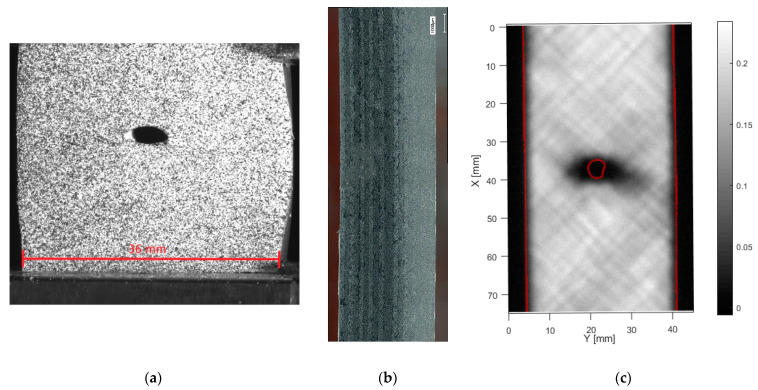
Compressive buckling of specimen with layup [+45/−45]_5s_, (**a**) front view, (**b**) microscopy of side, (**c**) through transmission ultrasound (for high resolution, please refer to the online version of this article).

The samples with layup [+45/−45]_5s_ showed large nonlinear behavior before failure for both tensile and compressive loading. Figure 7 shows typical observations under tensile loading, while Figure 8 shows typical observations under compressive loading. OHT failure for the samples with layup [+45/−45]_5s_, Figure 7, shows a combination of crack growth and ductile failure. Before the failure, multiple cracks develop at the hole edge, as indicated in Figure 7a. However, these initial cracks do not necessarily grow to cause final failure. In Figure 7a,b, the same crack is indicated with a red circle. Since this crack appears to be the largest, one could expect this to cause a final fracture. Surprisingly, the crack causing load-bearing failure occurs from a different location. The main failure phenomenon is ply delamination. This is shown on the microscopic images. In Figure 7c the separation between the [−45] and [+45] plies, that each remained attached to one-half of the specimen. A limited amount of matrix cracks is observed. The extent of internal delaminations for this layup is shown in Figure 7d. It can be seen that the ply separations do not extend further than the center area.

OHC failure for the samples with layup [+45/−45]_5s_, Figure 8, could not be achieved. The samples buckled at about −100 MPa nominal stress. Figure 8a shows the sample deformation, which could be observed. To protect the test machine, and especially the clamp alignment, it was not attempted to fully break the samples. Therefore, the samples were unloaded and removed after the occurrence of buckling. Remarkably, side microscopy shows, apart from a permanent bend of the specimen, no signs of internal damage, Figure 8b. This is confirmed by ultrasound, Figure 8c, which only shows a limited amount of delamination around the hole edge. The absence of matrix cracking or delamination after such severe out-of-plane deformation is attributed to the ductility of the PA6 matrix combined with a strong fiber-matrix bond.

**Figure 9:**
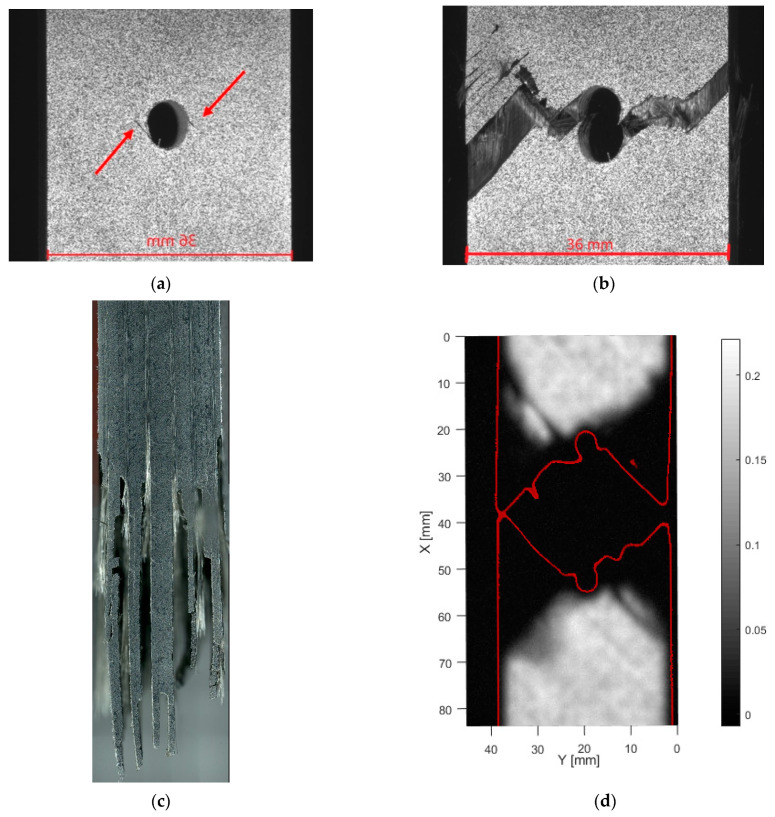
Tensile failure of [+45/0/−45/90]_3s_, (**a**) front view just before failure, (**b**) front view after failure (**c**) side microscopy, (**d**) through transmission ultrasound (for high resolution, please refer to the online version of this article).

**Figure 10:**
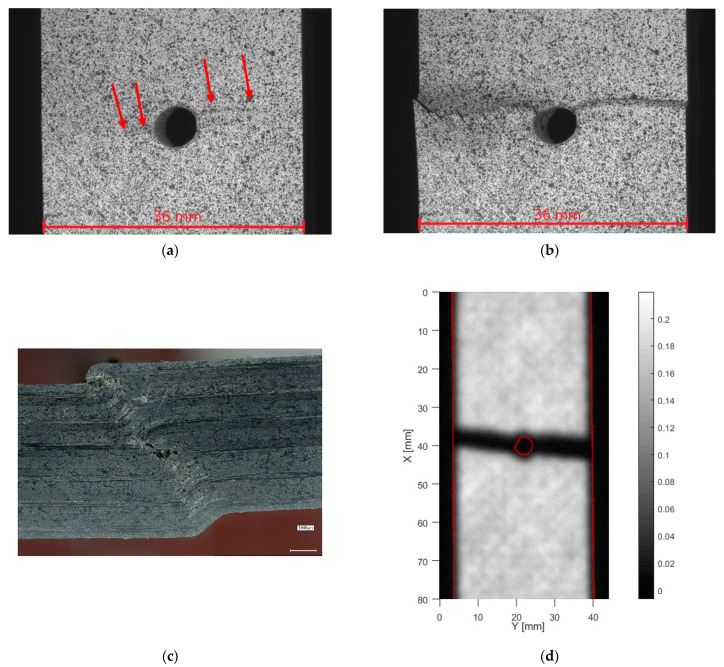
Compressive failure of [+45/0/−45/90]_3s_, (**a**) front view just before failure, (**b**) front view after kinking failure, (**c**) side microscopy, (**d**) through transmission ultrasound (for high resolution, please refer to the online version of this article).

Figure 9 and Figure 10 show the tensile and compressive failure for specimens with layup [+45/0/−45/90]_3s_. For the tensile case, Figure 9a,b, cracks are seen to develop along the +45 fiber direction of the top ply. These grow from the hole edge towards the edges of the sample until final failure. The specimen fails in a brittle way due to the presence of the 0 degree plies. Side microscopy, Figure 9c, shows a combination of the fracture phenomena observed in the previous layups. The [0] plies show fiber pull-out with delamination, [90] plies show matrix cracking and the [±45] plies failed in the typical shear-out failure. Associated with the failure of the latter plies is the internal delamination, as in Figure 9d.

Similar to [0/90]_5s_, the compressive failure of [+45/0/−45/90]_3s_ occurs in the form of kinking, Figure 10. Buckling was not observed during loading. However, it is noted that the specimens do not fracture in two pieces, as seen from the side view in Figure 10c. The kink band is also clearly visible in the ultrasound, Figure 10d. The delamination damage is contained within the kinking zone. Combining plies in all three primary directions can keep the plies from both sides of the specimen connected and attached, even after severe kink deformation. This allows the specimen to carry load after initial failure.

### 3.3. Evolution of Elastic Modulus and Permanent Strain

From the nominal stress–global strain of the LUR tests, both the evolution of permanent strain and elastic modulus are derived. Additionally, the strain field on the specimen surface after unloading indicates the locations and pattern of permanent deformation.

**Figure 11:**
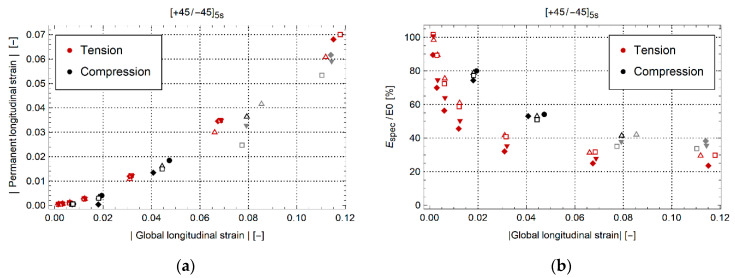
(**a**) Permanent strain and (**b**) evolution of normalized elastic modulus for [+45/−45]_5s_. Datapoints in gray are measured after buckling.

Figure 11 shows the evolution of permanent strain and modulus of the specimen with layup [+45/−45]_5s_. On the vertical axis, figures show the longitudinal specimen modulus ($E_{spec}$) normalized concerning the undamaged elastic modulus (E0) and longitudinal specimen permanent strain. One can clearly see that a large amount of permanent strain develops. In addition, the elastic modulus reduces with each load cycle up to about 30% of the original modulus for tension and 40% (before buckling) for compression. In figures, the same symbols are used for the same test. The data points in gray occurred after buckling. Between tensile and compression loading, the evolution of permanent strain is similar. The evolution of specimen elastic modulus is less steep for compression.

**Figure 12:**
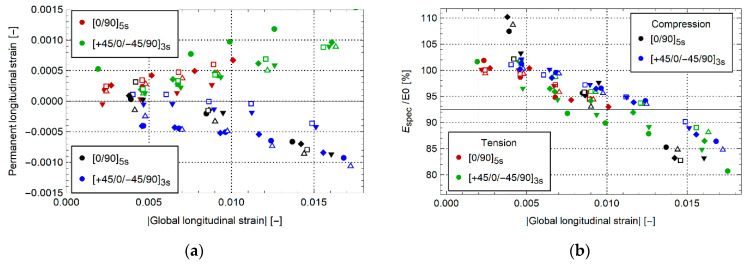
(**a**) Permanent strain and (**b**) normalized elastic modulus for [0/90]_5s_ and [+45/0/−45/90]_3s_.

Figure 12 shows the evolution of the permanent longitudinal strain and elastic modulus for [0/90]_5s_ and [+45/0/−45/90]_3s_. Though the nominal stress–global strain curve is quasilinear, both layups show a small evolution in the permanent global strain with a modulus reduction of about 20%. This suggests that, at least locally, some damage and plasticity occur in the specimen volume. Similar evolutions are shown between tension, compression, and per layup. Note the initially higher elastic modulus after the first unloading cycle in the compression data of Figure 12b. This is caused by variability in strain measurement at small strain magnitude. Despite this, both elastic modulus evolution and permanent strain evolution are similar between tension and compression.

**Figure 13:**
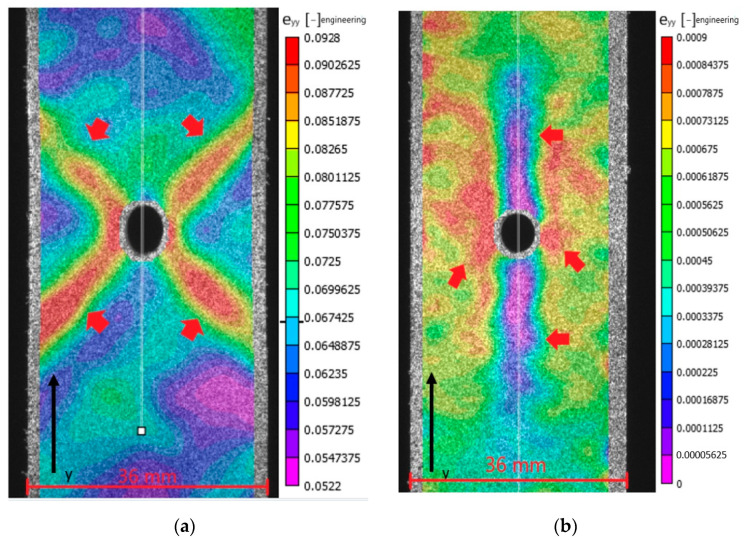
Permanent strain developed after unloading specimen (**a**) LUR-OHT with layup [+45/−45]_5s_ and (**b**) [0/90]_5s_ after the highest loading cycle before failure.

**Figure 14:**
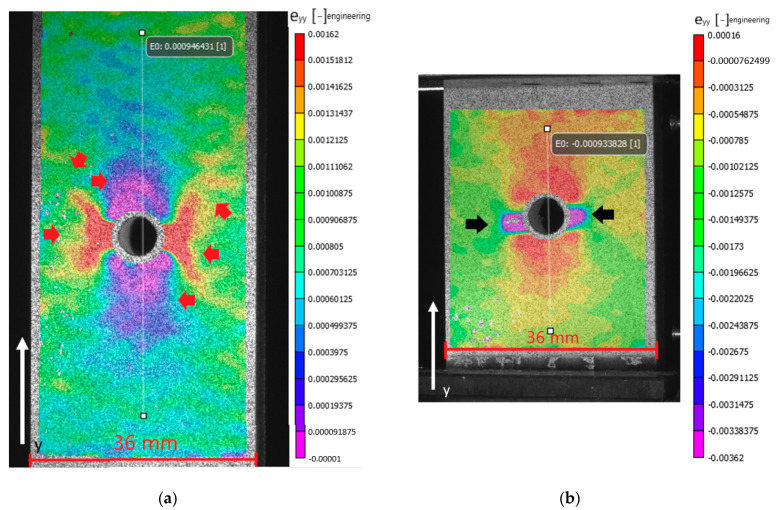
Permanent strain developed after unloading specimen (**a**) LUR-OHT and (**b**) LUR-OHC for the laminate with layup [+45/0/−45/90]_3s_ after the highest loading cycle before failure.

Although the global specimen behavior reveals the evolution of permanent strain and elastic modulus, it does not show where the permanent strain occurs. This can be identified from the surface strain fields obtained by DIC. Figure 13 and Figure 14 show the longitudinal strain on the specimen surface at unloading after either the last load cycle or just before buckling. Note that the scales associated with individual figures are different. This was done to provide the clearest view of the variation in strain for each figure. For the samples with layup [+45/−45]_5s_ X-shaped shear bands with high permanent strain develop. These meet at the side of the hole. This is illustrated in Figure 13a for the tensile case where the longitudinal strain is positive. For compression, the longitudinal strain has the same shape yet with negative strain values. For the samples with layup [0/90]_5s_, Figure 13b, a region of nearly zero longitudinal strain occurs above and below the hole center. To the left and right of the hole, a local region with permanent strain occurs. For the tensile case, the longitudinal strains are positive, while for the compressive case, these are negative. The region with larger permanent strain is limited to the immediate vicinity of the hole, which is in sharp contrast with layup [+45/−45]_5s_, where the region of permanent strain extends fully to the specimen edges.

The permanent strain pattern for the QI layup, Figure 14, shows a combination of the patterns from the [0/90]_5s_ and [+45/−45]_5s_ laminates. In the tensile case, Figure 14a, regions with nearly zero permanent strain are present above and below the hole, while left and right from the hole, a region with limited permanent strain develops. Further away from the hole, this region splits into two arms, extending similarly to the X-shaped pattern seen in the [+45/−45]_5s_ laminate. In contrast to the other laminates, the QI laminate in compression, Figure 14b, does not seem to show an inverse pattern as the tensile case. In fact, this seems more similar to the compressive case of the [0/90]_5s_ laminate. The authors attribute this difference to the different failure phenomena occurring for this laminate under tension and compression. In fact, in the compressive case, one can already see the initial growth of the kink band forming close to the hole, while it does not yet span the full specimen width.

## 4. Conclusions

In this work, open hole tension and open hole compression tests were executed on three laminates from UD glass/PA6 material. The laminates used are [0/90]_5s_, [+45/−45]_5s_ and [+45/−0/−45/90]_3s_. Both monotonic and loading–unloading–reloading experiments were executed. This allowed identifying global specimen behavior, developing permanent strain, and evolution of specimen elastic modulus until final failure.

The experimental results show the significant nonlinear behavior of open-hole specimens made from thermoplastic fiber-reinforced materials under both tensile and compressive loading. The specimens with layup [+45/−45]_5s_ showed similar behavior under both tension and compression. Degradation of the specimen elastic modulus up to 70% and permanent longitudinal strain of 7% is seen. The specimens with layup [0/90]_5s_ and [+45/0/−45/90]_3s_ show a maximum reduction of about 20% in elastic modulus. A very small amount of 0.1% permanent longitudinal strain is observed.

The identification of permanent deformation as well as the elastic modulus degradation of the samples before failure is particularly useful for engineers in mechanical design and to validate new material models for thermoplastic materials. Even more so, the availability of the entire strain field on the sample surface throughout the test can be used to verify and validate finite element simulations of open-hole tests for this material. A remarkable observation is that, while the evolution of permanent global strain and elastic modulus reduction between the tensile and compressive load cases is quite similar, the distribution of permanent strain development can be different. This indicates the necessity to include full-field strain measurements, as well as both tensile as compressive loading, in future experimental programs on composite mechanical behavior.

## Figures and Tables

**Table 1:** Overview of specimens and load types.

Load Type	Layup	Length (mm)	Width (mm)	Thickness (mm)	Hole Diameter (mm)	Actuator Speed (mm/min)
Monotonic tensile LUR tensile	[0/90]_5s_	250	36	5.2	6	2
	[+45/−45]_5s_	250	36	5.2	6	2
	[+45/0/−45/90]_3s_	250	36	6.2	6	2
Monotonic compression LUR compression	[0/90]_5s_	150	36	5.2	6	0.5–0.6
	[+45/−45]_5s_	150	36	5.2	6	0.5–0.6
	[+45/0/−45/90]_3s_	150	36	6.2	6	0.5–0.6

## Data Availability

The research data for this article is stored at Ghent University for a minimum of five years after publication. The data can be requested by the corresponding author.

## References

[1] Stewart R. (2011). Thermoplastic composites—Recyclable and fast to process. Reinf. Plast..

[2] Clay S.B., Knoth P.M. (2016). Experimental results of fatigue testing for calibration and validation of composite progressive damage analysis methods. J. Compos. Mater..

[3] Hinton M., Kaddour A. (2012). Triaxial test results for fibre-reinforced composites: The Second World-Wide Failure Exercise benchmark data. J. Compos. Mater..

[4] Kaddour A., Hinton M., Smith P., Li S. (2013). Mechanical properties and details of composite laminates for the test cases used in the third world-wide failure exercise. J. Compos. Mater..

[5] Mariatti M., Nasir M., Ismail H., Backlund J. (2004). Effect of Hole Drilling Techniques on Tensile Properties of Continuous Fiber Impregnated Thermoplastic (COFIT) Plain Weave Composites. J. Reinf. Plast. Compos..

[6] Brown N., Worrall C., Ogin S., Smith P. (2015). Investigation into the mechanical properties of thermoplastic composites containing holes machined by a thermally-assisted piercing (TAP) process. Adv. Manuf. Polym. Compos. Sci..

[7] El-Dessouky H., Saleh M., Gautam M., Han G., Scaife R., Potluri P. (2019). Tailored fibre placement of commingled carbon-thermoplastic fibres for notch-insensitive composites. Compos. Struct..

[8] Zhang H., Dickson A.N., Sheng Y., McGrail T., Dowling D.P., Wang C., Neville A., Yang D. (2020). Failure analysis of 3D printed woven composite plates with holes under tensile and shear loading. Compos. Part B Eng..

[9] Liu H., Falzon B.G., Li S., Tan W., Liu J., Chai H., Blackman B.R., Dear J.P. (2019). Compressive failure of woven fabric reinforced thermoplastic composites with an open-hole: An experimental and numerical study. Compos. Struct..

[10] Gavande V., Anand A. (2020). On the mechanical properties of continuous fiber reinforced thermoplastic composites realized through vacuum infusion. J. Compos. Mater..

[11] Vankan W.J., Tijs B.H., De Jong G.J., De Frel H.C., Singh N.K. (2016). Strength of notched and un-notched thermoplastic composite laminate in biaxial tension and compression. J. Compos. Mater..

[12] Niitsu G.T., Lopes C.M.A. (2013). Compressive Strength of Notched Poly(Phenylene Sulfide) Aerospace Composite: Influence of Fatigue and Environment. Appl. Compos. Mater..

[13] Malpot A., Touchard F., Bergamo S., Peyrac C., Montaudon R., Blumenfeld J.-B. (2018). Fatigue behaviour of open-hole samples and automotive mini-structures made of woven glass-fibre-reinforced polyamide 6,6. MATEC Web Conf..

[14] (2015). Ten Cate Advanced Composites.

[15] ASTM D5766/D5766M-11 (2014). Standard Test Method for Open-Hole Tensile Strength of Polymer Matrix Composite.

[16] ASTM D6484 (2014). Standard Test Method for Open-Hole Compressive Strength of Polymer Matrix Composite Laminates.

[17] Green B., Wisnom M., Hallett S. (2007). An experimental investigation into the tensile strength scaling of notched composites. Compos. Part A Appl. Sci. Manuf..

[18] (2020). VIC-3D Digital Image Correlation.

[19] (2020). LabVIEW.

